# Insect-based fish feed in decoupled aquaponic systems: Effect on lettuce production and resource use

**DOI:** 10.1371/journal.pone.0295811

**Published:** 2024-01-19

**Authors:** Sara Pinho, Margarida Meneses Leal, Christopher Shaw, Daniela Baganz, Gösta Baganz, Georg Staaks, Werner Kloas, Oliver Körner, Hendrik Monsees

**Affiliations:** 1 Leibniz-Institute of Vegetable and Ornamental Crops (IGZ), Grossbeeren, Germany; 2 Faculdade de Medicina Veterinária, Universidade de Lisboa, Lisboa, Portugal; 3 Leibniz Institute of Freshwater Ecology and Inland Fisheries, Berlin, Germany; 4 Albrecht Daniel Thaer-Institute of Agricultural and Horticultural Sciences, Berlin, Germany; 5 Institute of Biology, Humboldt University, Berlin, Germany; University of Eldoret, KENYA

## Abstract

The utilisation of insect meal-based fish feed as a substitute for conventional fish meal-based fish feed is considered as a promising innovative alternative to boost circularity in aquaculture and aquaponics. Basic research on its use in aquaponics is limited. So far, no reports on the effects of fish waste water, derived from a recirculating aquaculture system using Black Soldier Fly (BSF) meal-based diets, were available on the growth performance of lettuce. Therefore, this study aimed to compare the effect of reusing fish waste water from tilapia culture (as a base for the nutrient solution) fed with a fish meal-based diet (FM) and a BSF meal-based diet on resource use and lettuce growth in decoupled aquaponic systems. A conventional hydroponics nutrient solution (HP) served as control, and inorganic fertilisers were added to all nutrient solutions to reach comparable target concentrations. The experiment was conducted in a controlled climate chamber in nine separate hydroponics units, three per treatment. Lettuce fresh and dry weight, number of leaves, relative leaf chlorophyll concentration, water consumption, and the usage of inorganic fertilisers were measured. Micro- and macronutrients in the nutrient solutions were monitored in time series. Similar lettuce yield was seen in all treatments, with no significant effects on fresh and dry weight, the number of leaves, and relative chlorophyll values. Water use per plant was also similar between treatments, while the amount of total inorganic fertiliser required was 32% lower in FM and BSF compared to HP. Higher sodium concentrations were found in the FM nutrient solutions compared to BSF and HP. The results confirm that BSF-based diet is a promising alternative to FM-based diet in aquaponics with no negative effects on lettuce growth. Additionally, BSF-based diet might be beneficial in intensive, professional aquaponics applications due to the lower sodium concentration in the nutrient solution.

## Introduction

Aquaponics is a food production system that reuses nutrient-rich water from closed aquaculture systems for plant nutrition and irrigation [1–5]. Such a system combines aquatic animal production, usually fish in filter-based recirculating aquaculture systems (RAS), with soilless plant production in hydroponic systems [4, 6]. Aquaponics is based on sharing resources between the RAS and hydroponics units, and the degree of resource sharing depends on how the system is operated and the units are connected [1]. In decoupled aquaponic systems, the units are partially connected with a unidirectional flow of water and nutrients from RAS to hydroponics [1, 7]. As a result, decoupled systems allow for managing and controlling the environmental conditions at optimal levels for all organisms in every unit, and thus, high yield and efficiency of resource use are achieved [8–10]. While plant evapotranspiration processes drive water flow in decoupled aquaponic systems [11], nutrients are primarily input via fish feed [12, 13], and complementing the fish waste water with missing nutrients for plant production is a common practice [8, 14, 15]. Thereby special attention must be paid to the fish feed composition, as it affects the overall availability of nutrients in the system [13, 16] and the environmental impacts of the food production process [17, 18].

Fish feed relies on the use of fish meals as a key protein ingredient for fish production in intensive systems, such as RAS and aquaponics [12, 19] and might affect the overall sustainability in a negative way. Fish meal is commonly considered as a "gold standard ingredient" for feed formulation due to its high palatability and digestibility rates, balanced amino acid profile, and low or null anti-nutritional factors for most commercially farmed fish species [20]. The problem with using fish meal is the overexploitation of wild forage fish stocks for its production and the increase in prices [19, 21, 22]. Additionally, from an aquaponics point of view, fish meal-based fish feed reportedly results in undesirable sodium accumulation in the water, which is not beneficial for most freshwater crops [12]. Several studies have tested alternative protein ingredients to substitute fish meals in aquaculture. For example, animal by-products, plant-based, and insect-based meals have the potential to provide at least partially the protein required by Nile tilapia (*Oreochromis niloticus*) [22–24], one of the most produced fish worldwide [25]. However, non-insect-based, fishmeal-free diets do not generally result in a significant decrease in environmental impact compared to the use of biotic resources [26].

Insect meals have been presented as a sustainable alternative ingredient for the future of fish feed [27]. The most promising insect meal for fish diet is produced from Black Soldier Fly (BSF), *Hermetia illucens*, due to its high content of protein, lipids, minerals, and similar essential amino acid patterns to fish meal [28–30]. Recent studies reported BSF meal is easily digested by tilapia, improving feed use efficiency and availability of nutrients in the water [16, 31–34]. Moreover, BSF can be fed by a vast range of substrates (e.g. food waste, municipal sewage sludge, and fish sludge), making its production more eco-friendly compared to other animal by-products meals [35–38]. Considering that BSF can be produced by reusing the fish sludge discharged from the RAS unit, feeding the fish with BSF meal-based feed would boost aquaponics circularity [24] and hence the sustainability of the system.

Despite the benefits of BSF meal mentioned above, the research on its use in aquaponics, particularly in decoupled aquaponics, is limited. Some authors have been investigating the addition of BSF frass, a mineral-rich by-product of BSF production, as a source of supplementary nutrients in aquaponics [39, 40]. While others use BSF meal in fish feed, focusing on evaluating fish growth and nutrient release in RAS and discussing these results from the perspective of decoupled aquaponics production [16, 24, 41]. Until today, no scientific research was conducted and reported on reusing BSF meal-based fish waste water in the hydroponics unit of a decoupled aquaponic system. Therefore, this study aimed to compare the effect of using fish waste water (as a base for the nutrient solution) from Nile tilapia culture fed with fish meal-based diet (FM) and BSF meal-based diet on lettuce (*Lactuca sativa*) growth and resource use (water and inorganic fertiliser) in decoupled aquaponic systems. A conventional hydroponics nutrient solution (HP, control) served as a control group. Lettuce was chosen as the target crop since it is one of the main plants scientifically and commercially produced in aquaponics [4].

## Material and methods

### Experimental design and setup

The experiment was conducted at the Leibniz Institute of Freshwater Ecology and Inland Fisheries (IGB, Berlin, Germany) for 35 days in a climate-controlled growth chamber with a target room temperature of 18°C and relative humidity of 60% controlled by an integrated cooling system. The chamber hosted nine experimental tents (Royal Room C120S, 120x60x180 cm), each equipped with a hydroponics AeroFlo10 unit (Growstream®, GHE) and a light-emitting diode (LED) lighting system. The hydroponics unit consisted of two gutters (110 x 50 x 50 cm) with five plant-sites each, connected to a 45 L nutrient solution reservoir by a pump (compactON 1000, Eheim GmbH, Germany). The nutrient solution was constantly oxygenated using compressed air and air stone diffusers. The lighting system composed of one LED lamp (SANlight Q4WL S2.1 Gen2, 165W) positioned 90 cm to the top of the hydroponic unit and programmed to supply 91 μmol m^-2^ s^-1^ photosynthetic photon flux density (PPFD) at plant level for 12h each day. Digital timers (LOGILINK ET0007 timer, digital, max 1800 W) automatically switched the lights on in every tent at 6 am and off at 6 pm. In addition, an automated fan (PK125 EC-TC Prima Klima) was introduced in every tent to mitigate possible lighting heating, with a target air temperature of 22°C and humidity of 60%, using minimum speed of 10% and maximum speed of 20%. Air temperature and relative humidity in the tents were monitored by data loggers (HOBO UX100-011).

**Fig 1:**
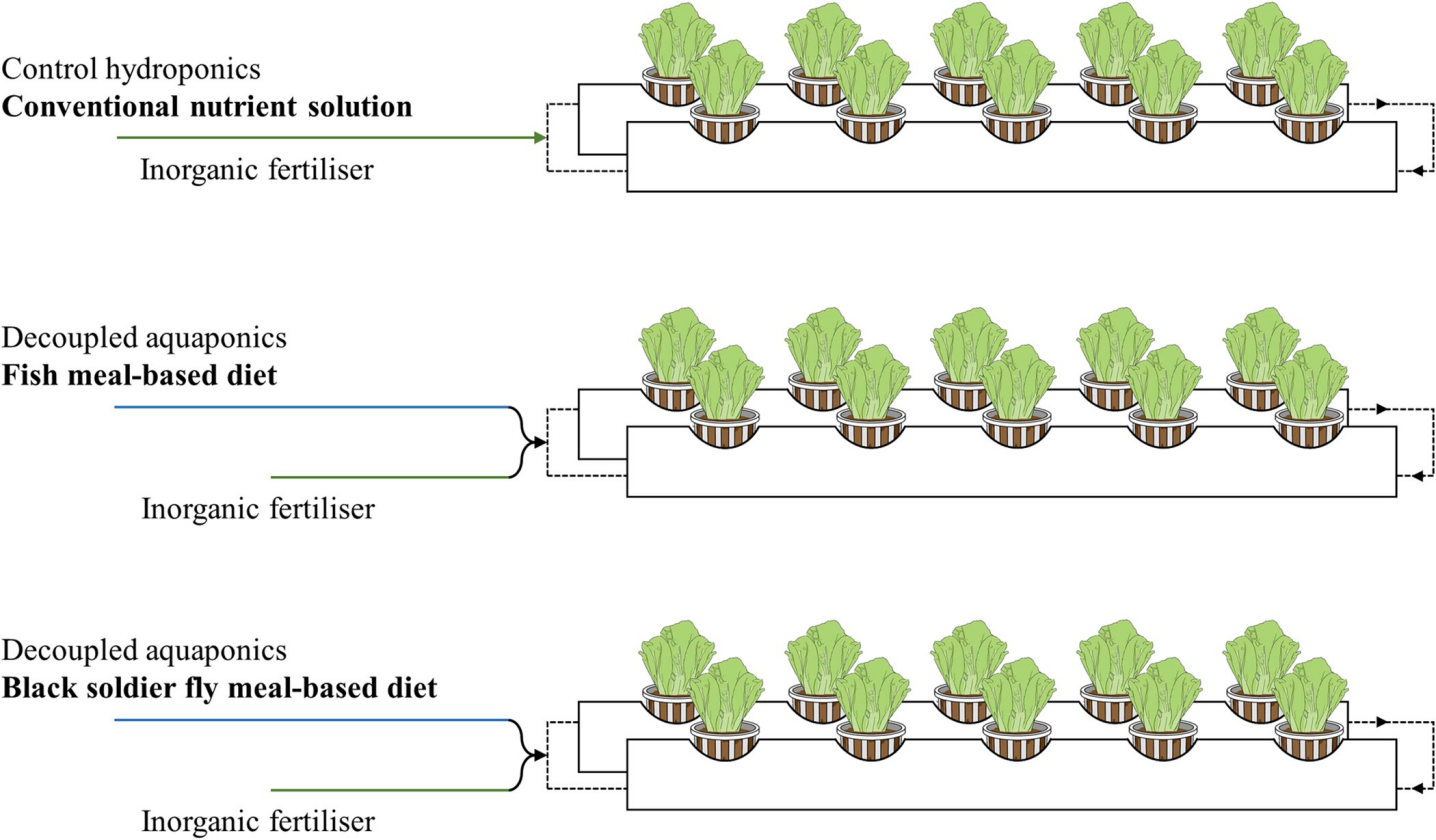
Representation of the experimental design. Lettuce production during 35 days under three nutrient solution sources: conventional hydroponics solution (HP, control) and fish waste water solutions from tilapia culture fed with fish meal-based diet (FM) and Black Soldier Fly meal-based diet (BSF). All treatments were tested in triplicate, i.e. nine experimental units were used.

A completely randomised experiment was designed to evaluate lettuce growth responses to three nutrient solution sources: conventional hydroponics solution (HP, control) and fish waste water solutions from tilapia culture fed with FM-based diet and BSF meal-based diet (Fig 1). All treatments were tested in triplicate using the nine experimental units. This study did not require an application for approval according to communication with the Ethics Committee of the Landesamt für Gesundheit und Soziales (LAGeSo), Berlin.

The fish water was taken from six experimental recirculating aquaculture systems (RAS) at IGB. The RAS were stocked with juvenile Nile tilapia (*Oreochromis niloticus*) that were reared for 56 days on two experimental diets differing by the type of protein ingredient: an entirely FM-based diet and an entirely BSF meal-based diet. This choice of diets allowed us to investigate the potential benefits and limitations of these specific fish diet protein ingredients in the context of lettuce production in decoupled aquaponics. The diets were formulated and produced to meet the nutritional requirements of Nile tilapia (Table 1). As the present study is focused on lettuce performance (i.e. the hydroponics part of the decoupled aquaponic system), a brief summary of the fish rearing conditions and results is presented in S1 Appendix.

**Table 1:** Formulation and proximate analysis of experimental diets: Fish meal-based diet (FM) and Black Soldier Fly meal-based diet (BSF).

*Ingredients*	Diet (g kg^-^^*1*^)	
	FM	BSF
*Fish meal* ^*1*^	52.6	-
*Black soldier fly meal* ^*2*^	-	52.6
*Wheat bran* ^*3*^	29.5	29.5
*Corn meal* ^*4*^	10.1	10.1
*Fish oil* ^*5*^	6.1	6.1
*Dicalcium phosphate* ^*6*^	1.2	1.2
*Vitamin and mineral premix* ^*7*^	0.5	0.5
***Proximate composition*** *		
*Crude protein*	40.55	37.65
*Crude fat*	12.25	8.00
*Ash*	12.05	7.65
*Crude fibre*	2.40	6.60
*Phosphorus*	1.93	1.25
*Dry mass*	94.05	93.80

^1^ Bioceval GmbH & Co. KG, Cuxhaven

^2^ Hermetia Baruth GmbH, Baruth/Mark

^3^ Hoveler Pferdefutter GmbH, Munster

^4^ M + M Baits, Neuenkirchen-Vorden

^5^ Scheidler GmbH, Eystrup

^6^ Th. Geyer GmbH & Co. KG, Berlin

^7^ Bionic Natura GmbH & Co. KG, Munchweiler an der Rodalb

* Analysed by Landesamtliche Untersuchungs- und Forschungsastalt Speyer (LUFA Speyer), Obere Langgasse 40, 67346 Speyer.

### Lettuce material and growth conditions

Lettuce (*Lactuca sativa* L., Aquino RZ cv., Rijk Zwaan; De Lier, The Netherlands) seeds were sown in stone-wool cubes (4 cm, Rockwool®, Grodan,The Netherlands) and germinated during 14 days under controlled environmental conditions in a climate chamber (temperature: 18°C, PPFD: 250 μmol m^-2^ s^-1^, and humidity: 90% for 2 days and 60% for the remaining 12 days). Then, ten plants were allocated in every hydroponics unit at a plant density of 18 plants m^-2^ and produced for 35 days until harvest. The plants were irrigated with a continuous flow (1.5 L min^-1^, over 24h) and had a light interval of 12h of light followed by 12h of darkness.

Before allocating the plants, the reservoir of each hydroponics unit was filled with the specific treatment nutrient solution. The solutions were changed twice during the experiment, on the 14^th^ and 27^th^ day. On both occasions, the "old" nutrient solution was pumped out and replaced with a "fresh" nutrient solution specific to the treatment being tested. The formulation and preparation of the nutrient solution were similar for all reservoir filling procedures (on day 0 and during the water exchange process on days 14 and 27).

### Nutrient solutions preparation

Two days prior to each reservoir filling procedure, samples of tap water and fish waste water from both aquaponics treatments (FM and BSF) were analysed for their nutrient concentrations using inductively coupled plasma-optical emission spectrometry (ICP-OES) and continuous flow analysis (CFA) (Table 2). The results of these analyses were used to calculate the amount of inorganic fertiliser to be added in the form of stock solutions so that all solutions had a similar nutrient profile, targeting the Howard-Resh recipe for hydroponics lettuce nutrient solution (165 NO_3_-N, 15 NH_4_-N, 50 P, 210 K, 45 Mg, 190 Ca, 65 S, 4 Fe, 0.1 Zn, 0.5 B, 0.5 Mn, 0.1 Cu, 0.05 Mo, 0 Na, Si and Cl–all values in mg L^-1^).

**Table 2:** Nutrient concentrations in mg L^-1^ measured in samples of tap water and fish waste water from both aquaponics treatments, collected over three sampling periods (day 0, 14 and 27).

*Nutrients*	Day 0			Day 14			Day 27		
	Tap water*	FM	BSF	Tap water*	FM	BSF	Tap wate*	FM	BSF
NO_3_-N	1.3	36.0	32.6	1.1	47.1	45.3	1.2	40.9	37.8
NH_4_-N	0.01	0.39	0.29	0.01	0.50	0.29	0.11	0.19	0.20
P	0.01	3.0	2.3	0.01	4.7	3.4	0.01	2.8	2.4
K	5	13	18	5.6	15	24	6.5	13.3	19
Ca	105	126	127	123	116	118	115	115	117
Mg	11.5	15.7	17.5	13.2	16.0	18.0	12.9	15.2	16.9
S	51	68	67	60	64	62	55	63	61
Fe	<0.01	0.02	0.02	<0.01	<0.01	0.02	<0.01	<0.01	0.01
B	0.07	0.08	0.08	0.08	0.07	0.06	0.08	0.08	0.07
Mn	<0.01	<0.01	0.02	<0.01	0.09	<0.01	<0.01	<0.01	<0.01
Cu	0.04	0.02	0.02	0.31	0.02	0.03	0.01	0.01	0.02
Zn	0.09	0.02	0.05	0.52	0.03	0.04	0.02	<0.01	0.03
Na	42	50	41	42	56	44	40	50	42
Si	6.4	7.1	7.4	7.0	6.9	7.1	7.0	7.0	7.0
Al	<0.01	<0.01	<0.01	<0.01	0.02	0.02	0.02	0.02	0.02

* aa in distilled water, v:v, 50:50, for preparing the control hydroponics nutrient solution. FM: fish meal-based diet waste water. BSF: Black Soldier Fly meal-based diet waste water.

For the control treatment (HP), the nutrient solution was prepared with 50% tap water and 50% distilled water and added nutrients. The FM treatment nutrient solution was prepared with waste water from the RAS rearing tilapia fed with FM-based diet plus the addition inorganic fertilisers. For the BSF treatment, nutrient solution was prepared with waste water from the RAS rearing tilapia fed with a BSF meal-based diet plus inorganic fertiliser. 135 L of fish waste water used in the hydroponics units of each aquaponics treatment were collected from three RAS-corresponding treatments (45 L per RAS replicate) three times during the experiment: prior to day 0, day 14, and finally, day 27. The fish waste water from all replicates was mixed for each treatment and stored individually in tanks (for FM and BSF separately) for further preparation of the nutrient solutions

The amount of mineral nutrients added to the water sources as fertiliser mix was calculated using Hydrobuddy v1.91 program, formulating A+B concentrated stock solutions composed of multiple nutrients. For that, we fed the Hydrobuddy program with information on the target nutrient profile in the final solution, the nutrient profile of the water sources (Table 2), and the composition of the fertilisers available at IGB. For stock solution A, the following fertilisers were used: Potassium Nitrate–KNO_3_, Ammonium nitrate–NH_4_NO_3_, Iron EDDHA–FeEDDHA, Yara Calcium Nitrate–Yara_Ca(NO_3_)_2_, Magnesium Nitrate Solution–Mg(NO_3_)_2_.6H_2_0; for stock solution B: Potassium Monobasic Phosphate–KH_2_PO_4_, Boric Acid–H_3_BO_3_, Zinc Sulfate (dihydrate)–ZnSO_4_.2H_2_0, Manganese Sulfate (monohydrate)–MnSO_4_.H_2_0, Copper Sulfate (pentahydrate)–CuSO_4_5H_2_0. 135 L of solution per treatment was considered in every reservoir filling procedure, and the concentration factor and volume of each A or B stock solution were set to 100 and 1.35 L, respectively. The water parameters for the final nutrient solution were set at EC 1.6 mS cm^-1^ and pH 6.0.

Using the outputs from Hydrobuddy, the separate fertilisers were weighted and added to the respective six beakers (A and B stock solutions *vs* three treatments). The beakers were filled with 1 L distilled water and stirred until all the salts were dissolved. Each beaker was then filled with distilled water to a final volume of 1.35 L, representing a 100-fold concentration (i.e. 1% of the nutrient solution volume). Afterwards, the stock solutions A and B were added to the HP, FM and BSF tanks and adjustments were made to reach a pH value between 6.1 and 6.3, using nitric acid or sodium hydroxide to decrease or increase the pH, respectively. Once all the nutrient solutions were ready, samples were collected in duplicate for analysing nutrient content by ICP-OES and CFA methods. Then, nutrient solutions were distributed from the 135 L tanks to the 45 L replicate reservoirs.

### Plant measurements

Relative leaf chlorophyll concentration (soil plant analysis development—SPAD value) was measured in every lettuce plant one week before the harvesting (day 28) using a Chlorophyll Meter SPAD-502Plus device (Konica Minolta, Japan). The SPAD value was taken in five leaves per plant. The device calculated automatically the mean SPAD value of the five leaves resulting in the mean SPAD value of the lettuce plant.

At the end of the experiment, all lettuce heads were harvested by cutting the organ directly above the stone-wool cube. Immediately after harvesting, lettuce heads were weighted individually using precision balance (Kern EWJ, Reichelt elektronik GmbH, Sande, Germany) to determine the fresh weight. Then, four lettuce heads per replicate were randomly selected to count the number of leaves and prepare subsamples for further analyses. The subsamples were prepared by sampling one-quarter of each selected plant, weighted, placed into plastic bags, and stored at -80°C. Afterwards, the subsamples were freeze-dried for at least 72 h (Christ Alpha 1–4, Christ; Osterode, Germany). Every subsample was weighted again using analytical balance (Sartorius CP225D, Sartorius AG, Goettingen, Germany) to determine the dry weight and to calculate it for the whole lettuce head (multiplying sample value by 4).

### Water measurements and resource use

The abiotic parameters of the nutrient solutions, pH, temperature, electrical conductivity (EC), and dissolved oxygen (DO) concentration, were measured in all replicates on all weekdays during the experimental period. The pH, EC and temperature of the nutrient solutions were measured with the Hach Lange HQ40d probe. The DO concentration was measured using a dissolved oxygen meter, OxyGuard Handy Polaris. Water consumption was measured three times at every water exchange moment and at the end of the experiment. These data were used to estimate the volume of water needed per plant per day (V_w_) by summing the differences of the initial (V_0_) and final (V_f_) volume of the nutrient solution reservoirs of every replicate in the three measuring points (i = on day 14, 27 and 35): V_w_ = ∑(V0,i−V_f,i_).

For nutrient analysis, all water samples were collected in duplicate, filtered with a 0.2 μm cellulose acetate membrane filter (GE Healthcare, United Kingdom), preserved by adding 150 μL of 2M of hydrochloric acid (HCl) to 12 mL filtered sample and stored at 4°C for subsequent analysis. The samples were analysed right after the experiment ended. The concentrations of phosphorus (P), potassium (K), calcium (Ca), magnesium (Mg), sulphur (S), iron (Fe), boron (B), manganese (Mn), copper (Cu), zinc (Zn), sodium (Na), silicon (Si) and aluminium (Al) were determined by the Inductively Coupled Plasma-Optical Emission Spectroscopy (ICP-OES) using the Thermo Scientific iCAP 7400 ICP-OES (Thermo Fisher Scientific Inc., USA). The continuous flow analysis (CFA) was performed using the FSR Seal High Resolution AA3 chemical analyser (Seal Analytical, Germany) to determine nitrate (NO_3_-N) and ammonium (NH_4_-N) concentrations. In addition to the samples of tap water and fish waste water (Table 2), water samples were collected for characterising the fresh nutrient solutions on days 0, 15 and 26, and every week after the water exchange procedure in all replicates on days 6, 21, and 33.

The amount of inorganic fertiliser (IF, in grams) needed to prepare a suitable nutrient solution for lettuce production was estimated by averaging the differences in the initial and final amount of all nutrients per treatment at all measuring points (i.e. on days 14, 27, and 35), using the following equation: IF_j_ = ∑ [(n_0,i_ · V_0,i_)–(n_f,i_ · V_f,i_)] / N_mp_; where, IF_j_ = amount of inorganic fertiliser in the *j* treatment, n_0,i_ = concentration of each nutrient (n) in the initial water source (based on Table 2 and considering that tap water was further diluted in distilled water, v:v, 50:50, for preparing the control hydroponics nutrient solution), n_f,i_ = final concentration of each nutrient (n) in the fresh solution (after adding the inorganic fertiliser), and N_mp_ = number of measuring points, i.e. reservoir filling procedures (N_mp_ = 3). Using the results obtained from the equation, the proportion between the total amount of inorganic fertiliser needed to produce a control hydroponic nutrient solution, compared to FM and BSF, was calculated. The average percentage difference between the aquaponics and control HP treatments for every macronutrient (NO_3_-N, NH_4_-N, K, P, Ca, Mg, and S) in the form of inorganic fertiliser was calculated by: Dif_IF_n_ (%) = ((mean(IF_n,j_)—mean(IF_n,control_)) / mean(IF_n,control_)) * 100; where Dif_IF_n_ is the percentage difference of the amount of inorganic fertiliser for the specific macronutrient *n*, IF_n,j_ is the amount (g) of the inorganic fertiliser for the specific macronutrient *n* in the *j* treatment, and IF_n,control_ is the amount (g) of the inorganic fertiliser for the specific macronutrient *n* in the control HP treatment.

### Data processing and statistical analysis

Data were pre-processed using Microsoft Excel and analysed in MATLAB (ver. 2020b, "Statistics and Machine Learning" toolbox, TheMathWorks Inc., Portola, CA, USA). Shapiro-Wilk’s and Levene’s tests were applied to all data to check the normality and homogeneity of variances, respectively. Once the assumptions were met, a one-way ANOVA was used to determine whether the treatment affected the measured response variables: lettuce growth parameters, SPAD values, and water consumption. The data on the amount of inorganic fertiliser was not Gauss-distributed, thus it was subjected to nonparametric Kruskal-Wallis and Dunn’s tests to observe whether the medians varied significantly between the different treatments. All data were analysed at 5% significance level. Descriptive statistics were used to present the results for abiotic conditions and nutrient concentrations in nutrient solutions. In the tables, values represent means ± standard deviations.

## Results

The use of waste water from tilapia culture fed with a FM-based diet and BSF meal-based diet resulted in similar lettuce yields compared to conventional hydroponic nutrient solution (HP, control treatment). As shown in Table 3, the lettuce fresh and dry weight, the number of leaves and SPAD values were statistically not significant different among all treatments (*α* 0.05). The same was found for water use per plant (*p* > 0.05, Table 3). The amount of fertiliser needed, however, differed between treatments, being approximately 32% lower in FM and BSF compared to HP. Table 4 shows the average percentage difference between the aquaponics and control HP treatments for every macronutrient (NO_3_-N, NH_4_-N, K, P, Ca, Mg, and S) added in the form of inorganic fertiliser.

**Table 3:** Growth parameters, SPAD values, and water use by lettuce grown in the conventional hydroponic system (HP) compared to decoupled aquaponic systems reusing fish waste water from tilapia culture fed with fish meal-based diet (FM) and Black Soldier Fly meal-based diet (BSF).

*Parameters*	HP	FM	BSF	*p*-value
Fresh weight (g)	99.10 ± 3.83	98.60 ± 5.33	96.87 ± 5.14	0.8437*
Dry weight (g)	4.24 ± 0.14	4.38 ± 0.27	4.36 ± 0.16	0.6492*
Number of leaves (n)	116 ± 2	120 ± 3	120 ± 5	0.4156*
SPAD value (-)	28.53 ± 0.31	28.15 ± 0.40	27.77 ± 0.41	0.1200*
Water use (mL plant ^-1^ day ^-1^)	44.3 ± 8.4	35.3 ± 6.9	36.9 ± 16.7	0.6132*
Total inorganic fertiliser (g)	28.9 ± 0.04^a^	19.8 ± 0.06^b^	19.9 ± 0.06^b^	0.0390**

* One-way ANOVA test

** Kruskal-Wallis test, followed by post hoc Dunn’s test. Different letters indicate significant differences among treatments. All tests were performed at 5% significance level.

**Table 4:** Average percentage difference between the aquaponics and hydroponic control treatments for every macronutrient (NO_3_-N, NH_4_-N, K, P, Ca, Mg, and S) added in the form of inorganic fertiliser.

*Percentage difference* *	FM	BSF
Nitrate–NO_3_-N	- 29.21	- 25.85
Ammonia–NH_4_-N	- 49.33	- 48.05
Potassium–K	- 8.40	- 10.67
Phosphorus—P	- 2.55	- 0.17
Calcium—Ca	- 53.30	- 53.75
Magnesium—Mg	- 50.62	- 52.58
Sulphur—S	- 93.66	- 91.77
Total inorganic fertiliser	- 31.73	- 31.68

FM: fish meal-based diet waste water. BSF: Black Soldier Fly meal-based diet waste water.

* a(IF_n,j_)—mean(IF_n,control_)) / mean(IF_n,control_)) * 100; where Dif_IF_n_ is the percentage difference of the amount of inorganic fertiliser for the specific macronutrient *n*, IF_n,j_ is the amount (g) of the inorganic fertiliser for the specific macronutrient *n* in the *j* treatment, and IF_n,control_ is the amount (g) of the inorganic fertiliser for the specific macronutrient *n* in the control HP treatment.

**Fig 2:**
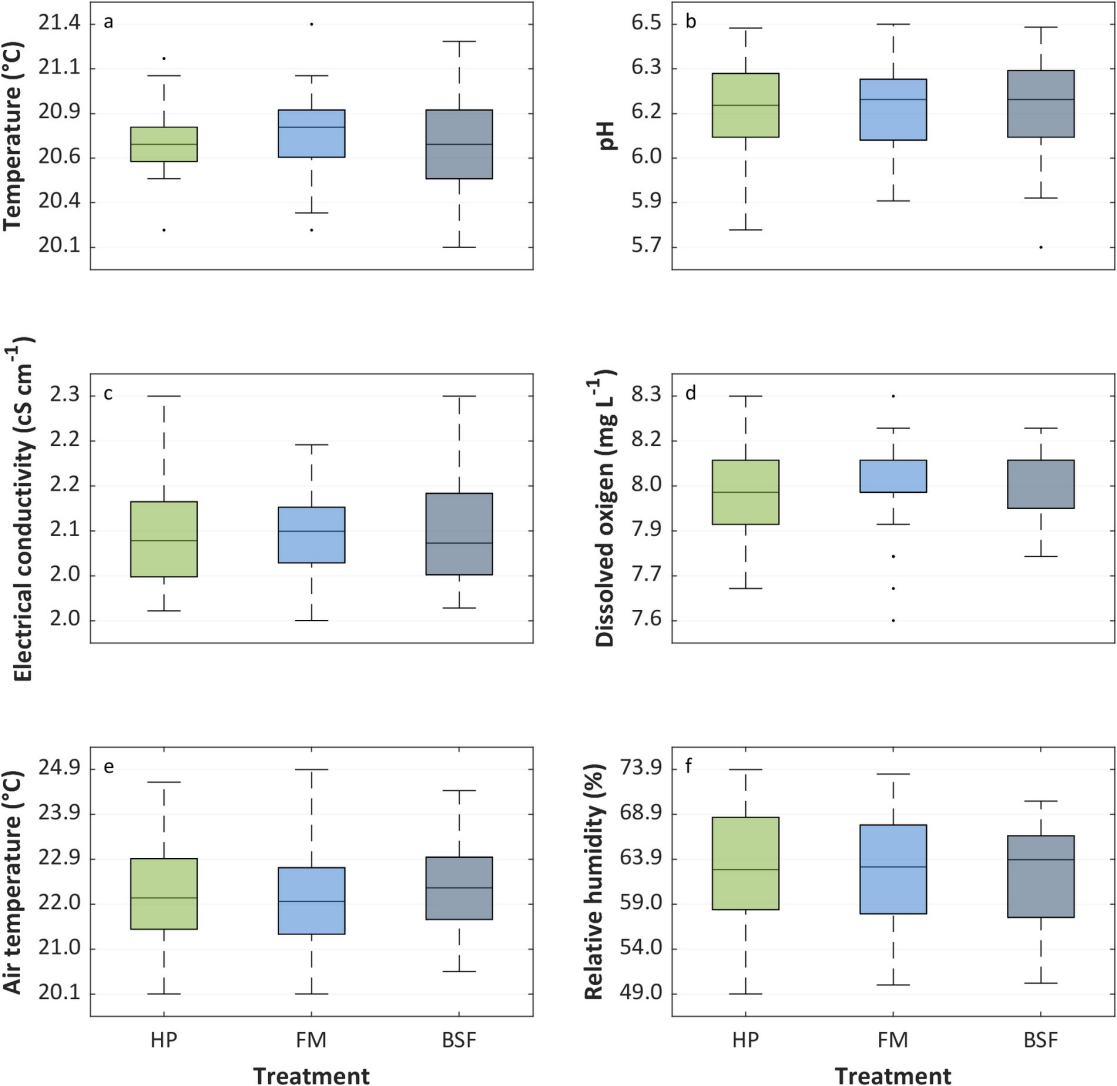
Boxplot overview of the measured nutrient solution parameter variables: (A) temperature, (B) pH, (C) electrical conductivity, and (D) dissolved oxygen; and abiotic environmental conditions: (E) air temperature and (F) relative humidity. Measurements were taken during the 35-day experimental period in the tents and in all nutrient solution replicates of the conventional hydroponics treatment (HP) and decoupled aquaponic systems reusing fish waste water from tilapia culture fed with fish meal-based diet (FM) and Black Soldier Fly meal-based diet (BSF).

The abiotic growth conditions in the tents were kept at the desired values in all tents, with mean air temperature and relative humidity of 22.1 ± 1.0°C and 62.7 ± 6.0%, respectively (Fig 2). The same occurs for the water quality condition, with values of electrical conductivity (EC), temperature, pH, and dissolved oxygen, in general, comparable between treatments (Fig 2).

For the nutrient concentration in the nutrient solutions over the experiment, no clear differences between the treatments were observed. The results of macronutrients (N, P, K, Ca, Mg and S) are presented in S1 Table and micronutrients (Fe, B, Mn, Cu, Zn, Na, Si, and Al) in S2 Table. In both tables, sampling days 0, 15 and 26 correspond to fresh nutrient solution. The Na concentrations showed remarkable differences, as the values were lower in HP, followed by BSF and higher in FM, with mean values of 25.8 ± 4.2 mg L^-1^ Na in the HP treatment, 46.0 ± 2.9 mg L^-1^ in the BSF treatment, and 59.4 ± 7.9 mg L^-1^ in the FM treatment.

## Discussion

The results of the present study indicate that waste water from tilapia culture in RAS fed with either a fish meal-based diet (FM) or a Black Soldier Fly meal-based diet (BSF) can be used as a basis for preparing a nutrient solution for lettuce cultivation, alternatively to conventional hydroponic nutrient solutions (HP). In terms of lettuce fresh and dry weight, number of leaves, and SPAD values, the FM and BSF treatments did not show any statistical difference. These results support the assumption that alternative feed ingredients such as BSF meal do not have any obvious effect on aquaponics lettuce production. Additionally, no differences in plant growth were found when comparing the aquaponics and control HP treatments. While some studies suggest that aquaponics can outperform hydroponics due to plant growth promoters from RAS, such as humic organic matter or beneficial microorganisms’ activity [14, 42], our findings are in line with most of the recent studies that have shown comparable plant growth in decoupled aquaponic and conventional hydroponic systems [10, 15, 43, 44]. Furthermore, all abiotic condition parameters were kept within acceptable limits for lettuce production [45, 46] without affecting plant growth and the subsequent results.

Achieving yields similar to those of stand-alone hydroponic systems is a positive and desirable outcome in aquaponics, especially considering that the primary reason for using aquaponic systems is to optimise resource use [47]. In fact vegetable production in decoupled aquaponics can contribute to reduced global warming potential and create a situation of turning the water footprint of the vegetable produce from a positive to a negative value [48]. Such benefits of aquaponics are seen in our study since the overall resource input was lower in aquaponics compared to hydroponics, with no effect of the fish feed composition (FM or BSF). Plant water use was similar in all treatments, suggesting that the nutrient solution source did not affect plant water uptake (supporting that there was no difference in other plant physiological parameters). However, as aquaponics relies mostly on the use of fish waste water to compensate for plant water uptake, while hydroponics relies on fresh water, it is reasonable to state that aquaponics using either FM or BSF-based fish feed (and thus fish waste water) is highly more efficient in terms of water use than hydroponics.

Concerning inorganic fertiliser use, the results are also relevant. The 32% reduction in inorganic fertiliser addition in both aquaponics nutrient solutions means that, in the conditions of our experiment, at least 32% of the overall nutrients required by lettuce are derived from fish water. The amount of saved inorganic fertiliser can even substantially be increased depending on e.g. system type and design, fish stocking density, amount of feed, and daily water exchange rate, as has been shown already by other studies [14, 49–51]. Inorganic fertiliser production contributes significantly to greenhouse gas emissions, particularly by releasing nitrous oxide during the manufacturing process [52]. If decoupled aquaponics are upscaled, the differences found in the total amount of inorganic fertiliser use could result in a considerable reduction in the environmental impact of soilless plant production. However, the predominant strategy for nutrient management in decoupled aquaponics is still the use of inorganic fertilisers.

One important side fact of BSF-fish waste water is that it could have significant implications for future nutrient management practices in aquaponics. BSF-fish waste water contains considerably less sodium compared to FM. Previous studies have raised concerns about the accumulation of NaCl in aquaponics [14, 53, 54]. For most of the greenhouse crops, the safe Na concentration in the hydroponic nutrient solution is around 50 mg L^-1^ [13]. Na concentrations can easily reach high levels in aquaponics depending on the recirculation method used (high, low, or drain-to-waste) in the hydroponic unit. It is known that salt stress reduces the growth, photosynthesis, and stomatal conductance of lettuce [55, 56]. However, based on the results found for the FM treatment (Table 3 and S2 Table) and previous studies [14, 15], 50 mg L^-1^ seems to be below a critical threshold for some lettuce cultivars. Different lettuce cultivars show different responses to salinity, leading to divergences in the findings regarding the effect of salinity on lettuce plant yield [57]. Adding to the differences created by the cultivar and genotype, environmental conditions (such as light intensity, temperature and relative humidity) and the cropping system are also responsible for influencing plant response to salinity stress. In addition to the lower Na concentration in the BSF treatment, chloride concentration may also be lower in the BSF-fish waste water due to the absence of fish meal, which contains certain levels of sodium chloride [13]. Chloride was not measured in this study, thus further statements regarding chloride are not possible. Nevertheless, the fact that a BSF-meal-based diet results in lower sodium concentration in the nutrient solution and potentially lower chloride concentration is valuable information for further research and the ongoing optimisation and improvement of aquaponic systems.

In general, aquaponic systems are already among the most efficient food production systems worldwide, especially when it comes to resource use efficiency. Using an insect meal-based diet for fish production in aquaponic systems would potentially bring aquaponics to a next level of circularity. Fish meal is a valuable resource and the pressure on this resource is increasing due to a rapid growth in aquaculture production [19, 21]. This leads to further increases in fish meal prices, but also to adverse environmental effects due to overfishing. Alternative protein sources, in particular insect-based meal, have the potential to fill the gap in fish meal and offer potential solutions to these issues. Insects are an effective sink for food waste, as well as residues of plant and animal production (e.g. fish sludge), making their sub-product a promising solution for fish-meal use issues. Our results indicated that no negative effects are expected regarding plant growth and resource use. Additionally, Devic et al. [58], Zhou et al. [59], and Shaw et al. [24] showed that substituting, at least partially, fish-meal with BSF-meal as the primary protein ingredient in fish diets does not have a negative impact on fish yield and feed use efficiency. Thus, reusing fish sludge as the primary source of feed for Black Soldier Fly production or simply using a BSF meal-based fish feed (if provided by professional feed producers) might have big beneficial effects on the overall circularity of aquaponic systems. Overall, the results of this study are very promising regarding the applicability of BSF meal-based fish feed in decoupled aquaponic systems, and we are confident that further research will pave the way for a wider application of this promising technology.

## Conclusion

The findings suggest that the use of insect-based fish feed in decoupled aquaponic systems is a resource-efficient alternative to produce lettuce without a reduction in yield. Using waste water from tilapia culture in RAS fed with fish meal-based diet (FM) and Black Soldier Fly meal-based diet (BSF) in decoupled aquaponic systems resulted in similar lettuce yields compared to conventional hydroponic nutrient solution (HP), as well as no adverse effects on lettuce growth were observed. Moreover, FM and BSF resulted in approximately 32% lower inorganic fertiliser uses compared to HP. The higher sodium concentrations in the FM nutrient solutions compared to BSF indicate that BSF-based diet is a promising alternative to FM-based diet in aquaponics, particularly for commercial, intensive applications. Our study supports the potential of using insect-based fish feed as an effective circular way to reduce the reliance on fish meal-based diets in aquaculture/aquaponic systems and the use of inorganic fertiliser for hydroponics systems.

## Supporting information

S1 Appendix Aquaculture subsystem. A brief description of the setup and management of the aquaculture subsystems, which generated the fish waste water used in the hydroponics units of the decoupled aquaponics systems, is provided.
(DOCX)

S1 Table Macronutrient concentrations (N, P, K, Ca, Mg, and S, mg L^-1^) in the nutrient solutions. (DOCX)

S2 Table Micronutrient concentrations (Fe, B, Mn, Cu, Zn, Na, Si, and Al, mg L^-1^) in the nutrient solutions. (DOCX)

## References

[1] Baganz GFM, Junge R, Portella MC, Goddek S, Keesman KJ, Baganz D, et al. The aquaponic principle—It is all about coupling. Reviews in Aquaculture. 2022;14: 252–264. doi: 10.1111/raq.12596

[2] Rakocy JE. Aquaponics-Integrating Fish and Plant Culture. In: Tidwell James, editor. Aquaculture Production Systems. Oxford, UK: Wiley-Blackwell; 2012. pp. 344–386. doi: 10.1002/9781118250105.ch14

[3] Krastanova M, Sirakov I, Ivanova-Kirilova S, Yarkov D, Orozova P. Aquaponic systems: biological and technological parameters. Biotechnology & Biotechnological Equipment. 2022;36: 305–316. doi: 10.1080/13102818.2022.2074892

[4] Yep B, Zheng Y. Aquaponic trends and challenges–A review. Journal of Cleaner Production. 2019;228: 1586–1599. doi: 10.1016/j.jclepro.2019.04.290

[5] Lennard W, Goddek S. Aquaponics: The Basics. In: Goddek S, Joyce A, Kotzen B, Burnell G, editors. Aquaponics Food Production Systems. Springer International Publishing; 2019. pp. 113–143. doi: doi.org/10.1007/978-3-030-15943-6_5

[6] Martinez-Cordova LR, Emerenciano MGC, Miranda-Baeza A, Pinho SM, Garibay-Valdez E, Martínez-Porchas M. Advancing toward a more integrated aquaculture with polyculture > aquaponics > biofloc technology > FLOCponics. Aquacult Int. 2022 [cited 9 Feb 2023]. doi: 10.1007/s10499-022-01016-0

[7] Kloas W, Groß R, Baganz D, Graupner J, Monsees H, Schmidt U, et al. A new concept for aquaponic systems to improve sustainability, increase productivity, and reduce environmental impacts. Aquaculture Environment Interactions. 2015;7: 179–192. doi: 10.3354/aei00146

[8] Monsees H, Kloas W, Wuertz S. Decoupled systems on trial: Eliminating bottlenecks to improve aquaponic processes. PLoS ONE. 2017;12: 1–18. doi: 10.1371/journal.pone.0183056 28957357 PMC5619720

[9] Lastiri DR, Geelen C, Cappon HJ, Rijnaarts HHMM, Baganz D, Kloas W, et al. Model-based management strategy for resource efficient design and operation of an aquaponic system. Aquacultural Engineering. 2018;83: 27–39. doi: 10.1016/j.aquaeng.2018.07.001

[10] Pinho SM, Lima JP, David LH, Oliveira MS, Goddek S, Carneiro DJ, et al. Decoupled FLOCponics systems as an alternative approach to reduce the protein level of tilapia juveniles’ diet in integrated agri-aquaculture production. Aquaculture. 2021;543: 736932. doi: 10.1016/j.aquaculture.2021.736932

[11] Goddek S, Körner O. A Fully integrated simulation mode of multi-loop aquaponics: a case study for system sizing in different environments. Agricultural systems. 2019;171: 143–154. 10.1016/j.agsy.2019.01.010

[12] Robaina L, Pirhonen J, Mente E, Sánchez J, Goosen N. Fish Diets in Aquaponics. In: Goddek S, Joyce A, Kotzen B, Burnell GM, editors. Aquaponics Food Production Systems: Combined Aquaculture and Hydroponic Production Technologies for the Future. Cham: Springer International Publishing; 2019. pp. 333–352. doi: 10.1007/978-3-030-15943-6_13

[13] Roy K, Kajgrova L, Mraz J. TILAFeed: A bio-based inventory for circular nutrients management and achieving bioeconomy in future aquaponics. New Biotechnology. 2022;70: 9–18. doi: 10.1016/j.nbt.2022.04.002 35395431

[14] Delaide B, Goddek S, Gott J, Soyeurt H, Jijakli MH. Lettuce (*Lactuca sativa L*. *var*. *Sucrine*) growth performance in complemented aquaponic solution outperforms hydroponics. Water (Switzerland). 2016;8: 1–11. doi: 10.3390/w8100467

[15] Monsees H, Suhl J, Paul M, Kloas W, Dannehl D, Würtz S. Lettuce (*Lactuca sativa*, variety Salanova) production in decoupled aquaponic systems: Same yield and similar quality as in conventional hydroponic systems but drastically reduced greenhouse gas emissions by saving inorganic fertilizer. PLOS ONE. 2019;14: e0218368. doi: 10.1371/journal.pone.0218368 31220125 PMC6586398

[16] Gebauer R, Brügmann A, Folorunso EA, Goldhammer T, Gebauer T, Schöning V, et al. Species- and diet-specific aquaculture wastewater nutrient profile: Implications for aquaponics and development of sustainable aquaponics diet. Aquaculture. 2023; 739307. doi: 10.1016/j.aquaculture.2023.739307

[17] David LH, Pinho SM, Agostinho F, Kimpara JM, Keesman KJ, Garcia F. Emergy synthesis for aquaculture: A review on its constraints and potentials. Reviews in Aquaculture. 2021;13: 1119–1138. doi: 10.1111/raq.12519

[18] Modahl IS, Brekke A. Environmental performance of insect protein: a case of LCA results for fish feed produced in Norway. SN Appl Sci. 2022;4: 183. doi: 10.1007/s42452-022-05065-1

[19] Agboola JO, Øverland M, Skrede A, Hansen JØ. Yeast as major protein-rich ingredient in aquafeeds: a review of the implications for aquaculture production. Reviews in Aquaculture. 2021;13: 949–970. doi: 10.1111/raq.12507

[20] Woodgate SL, Wan AHL, Hartnett F, Wilkinson RG, Davies SJ. The utilisation of European processed animal proteins as safe, sustainable and circular ingredients for global aquafeeds. Reviews in Aquaculture. 2022;14: 1572–1596. doi: 10.1111/raq.12663

[21] Cottrell RS, Blanchard JL, Halpern BS, Metian M, Froehlich HE. Global adoption of novel aquaculture feeds could substantially reduce forage fish demand by 2030. Nat Food. 2020;1: 301–308. doi: 10.1038/s43016-020-0078-x

[22] Mugwanya M, Dawood MAO, Kimera F, Sewilam H. Replacement of fish meal with fermented plant proteins in the aquafeed industry: A systematic review and meta-analysis. Reviews in Aquaculture. 2023;15: 62–88. doi: 10.1111/raq.12701

[23] Ng W-KK, Romano N. A review of the nutrition and feeding management of farmed tilapia throughout the culture cycle. Reviews in Aquaculture. 2013;5: 220–254. doi: 10.1111/raq.12014

[24] Shaw C, Knopf K, Kloas W. Fish Feeds in Aquaponics and Beyond: A Novel Concept to Evaluate Protein Sources in Diets for Circular Multitrophic Food Production Systems. Sustainability. 2022;14: 4064. doi: 10.3390/su14074064

[25] FAO. The State of World Fisheries and Aquaculture 2022—Towards Blue Transformation. Rome, Italy: Food and Agriculture Organization of the United Nations—FAO; 2022. doi: 10.4060/cc0461en

[26] Ghamkhar R, Hicks A. Comparative environmental impact assessment of aquafeed production_ Sustainability implications of forage fish meal and oil free diets. Resources, Conservation and Recycling. 2020;161: 104849. doi: 10.1016/j.resconrec.2020.104849

[27] Hua K, Cobcroft JM, Cole A, Condon K, Jerry DR, Mangott A, et al. The Future of Aquatic Protein: Implications for Protein Sources in Aquaculture Diets. One Earth. 2019;1: 316–329. doi: 10.1016/j.oneear.2019.10.018

[28] Adeoye AA, Akegbejo-Samsons Y, Fawole FJ, Davies SJ. Preliminary assessment of black soldier fly (*Hermetia illucens*) larval meal in the diet of African catfish (*Clarias gariepinus*): Impact on growth, body index, and hematological parameters. Journal of the World Aquaculture Society. 2020;51: 1024–1033. doi: 10.1111/jwas.12691

[29] Alfiko Y, Xie D, Astuti RT, Wong J, Wang L. Insects as a feed ingredient for fish culture: Status and trends. Aquaculture and Fisheries. 2022;7: 166–178. doi: 10.1016/j.aaf.2021.10.004

[30] Mohan K, Rajan DK, Muralisankar T, Ganesan AR, Sathishkumar P, Revathi N. Use of black soldier fly (*Hermetia illucens* L.) larvae meal in aquafeeds for a sustainable aquaculture industry: A review of past and future needs. Aquaculture. 2022;553: 738095. doi: 10.1016/j.aquaculture.2022.738095

[31] Limbu SM, Shoko AP, Ulotu EE, Luvanga SA, Munyi FM, John JO, et al. Black soldier fly (*Hermetia illucens*, L.) larvae meal improves growth performance, feed efficiency and economic returns of Nile tilapia (*Oreochromis niloticus*, L.) fry. Aquaculture, Fish and Fisheries. 2022;2: 167–178. doi: 10.1002/aff2.48

[32] Muin H, Taufek NM. Evaluation of growth performance, feed efficiency and nutrient digestibility of red hybrid tilapia fed dietary inclusion of black soldier fly larvae (*Hermetia illucens*). Aquaculture and Fisheries. 2022 [cited 13 Feb 2023]. doi: 10.1016/j.aaf.2022.09.006

[33] Aisyah HN, Athirah ZAR, Hanani WR, Arshad SS, Hassim HA, Nazarudin MF, et al. The effect of feeding black soldier fly larvae on growth performance, protein, and fat content of red hybrid tilapia (Oreochromis spp.). Vet World. 2022;15: 2453–2457. doi: 10.14202/vetworld.2022.2453-2457 36425138 PMC9682392

[34] Shaw C, Knopf K, Klatt L, Marin Arellano G, Kloas W. Closing Nutrient Cycles through the Use of System-Internal Resource Streams: Implications for Circular Multitrophic Food Production Systems and Aquaponic Feed Development. Sustainability. 2023;15: 7374. doi: 10.3390/su15097374

[35] Smetana S, Schmitt E, Mathys A. Sustainable use of *Hermetia illucens* insect biomass for feed and food: Attributional and consequential life cycle assessment. Resources, Conservation and Recycling. 2019;144: 285–296. doi: 10.1016/j.resconrec.2019.01.042

[36] Siddiqui SA, Ristow B, Rahayu T, Putra NS, Widya Yuwono N, Nisa’ K, et al. Black soldier fly larvae (BSFL) and their affinity for organic waste processing. Waste Management. 2022;140: 1–13. doi: 10.1016/j.wasman.2021.12.044 35030456

[37] Guidini Lopes I, Wiklicky V, Ermolaev E, Lalander C. Dynamics of black soldier fly larvae composting–Impact of substrate properties and rearing conditions on process efficiency. Waste Management. 2023;172: 25–32. doi: 10.1016/j.wasman.2023.08.045 37708809

[38] Arnone S, De Mei M, Petrazzuolo F, Musmeci S, Tonelli L, Salvicchi A, et al. Black soldier fly (*Hermetia illucens* L.) as a high-potential agent for bioconversion of municipal primary sewage sludge. Environ Sci Pollut Res Int. 2022;29: 64886–64901. doi: 10.1007/s11356-022-20250-w 35474429 PMC9481477

[39] Romano N, Fischer H, Powell A, Sinha AK, Islam S, Deb U, et al. Applications of Black Solider Fly (*Hermetia illucens*) Larvae Frass on Sweetpotato Slip Production, Mineral Content and Benefit-Cost Analysis. Agronomy. 2022;12: 928. doi: 10.3390/agronomy12040928

[40] Romano N, Islam S. Productivity and Elemental/Chlorophyll Composition of Collard Greens in an Aquaponic System at Different Combinations of Media and Black Soldier Fly (*Hermetia illucens*) Larvae Frass Supplementations. Aquaculture Research. 2023;2023: e3308537. doi: 10.1155/2023/3308537

[41] Shaw C, Knopf K, Kloas W. Toward Feeds for Circular Multitrophic Food Production Systems: Holistically Evaluating Growth Performance and Nutrient Excretion of African Catfish Fed Fish Meal-Free Diets in Comparison to Nile Tilapia. Sustainability. 2022;14: 14252. doi: 10.3390/su142114252

[42] Goddek S, Vermeulen T. Comparison of Lactuca sativa growth performance in conventional and RAS-based hydroponic systems. Aquacult Int. 2018;26: 1377–1386. doi: 10.1007/s10499-018-0293-8 30930556 PMC6405012

[43] Suhl J, Dannehl D, Kloas W, Baganz D, Jobs S, Scheibe G, et al. Advanced aquaponics: Evaluation of intensive tomato production in aquaponics vs. conventional hydroponics. Agricultural Water Management. 2016;178: 335–344. doi: 10.1016/j.agwat.2016.10.013

[44] Delaide B, Teerlinck S, Decombel A, Bleyaert P. Effect of wastewater from a pikeperch (*Sander lucioperca* L.) recirculated aquaculture system on hydroponic tomato production and quality. Agricultural Water Management. 2019;226: 105814. doi: 10.1016/j.agwat.2019.105814

[45] Mattson NS, Peters C. A Recipe for Hydroponic Success. Inside Grower. 2014; 16–19.

[46] Brechner M, Both AJ. Hydroponic Lettuce Handbook. 2013 [cited 17 Mar 2023]. Available: https://cpb-us-e1.wpmucdn.com/blogs.cornell.edu/dist/8/8824/files/2019/06/Cornell-CEA-Lettuce-Handbook-.pdf

[47] Ghamkhar R, Hartleb C, Wu F, Hicks A. Life cycle assessment of a cold weather aquaponic food production system. Journal of Cleaner Production. 2020;244: 118767. doi: 10.1016/j.jclepro.2019.118767

[48] Körner O, Bisbis MB, Baganz GFM, Baganz D, Staaks GBO, Monsees H, et al. Environmental impact assessment of local decoupled multi-loop aquaponics in an urban context. Journal of Cleaner Production. 2021;313: 127735. doi: 10.1016/J.JCLEPRO.2021.127735

[49] Dijkgraaf KH, Goddek S, Keesman KJ. Modelling innovative aquaponics farming in Kenya. Aquaculture International. 2019; 1395–1422. doi: 10.1007/s10499-019-00397-z

[50] Ezziddine M, Liltved H, Seljåsen R. Hydroponic Lettuce Cultivation Using Organic Nutrient Solution from Aerobic Digested Aquacultural Sludge. Agronomy. 2021;11: 1484. doi: 10.3390/agronomy11081484

[51] Tarigan NB, Goddek S, Keesman KJ. Explorative Study of Aquaponics Systems in Indonesia. Sustainability 2021, Vol 13, Page 12685. 2021;13: 12685. doi: 10.3390/SU132212685

[52] Menegat S, Ledo A, Tirado R. Greenhouse gas emissions from global production and use of nitrogen synthetic fertilisers in agriculture. Sci Rep. 2022;12: 14490. doi: 10.1038/s41598-022-18773-w 36008570 PMC9411506

[53] Beauchamp WR, Pickens JM, Sibley JL, Chappell JA, Martin NR, Newby AF. Salt Level in a Simulated Aquaponic System and Effects on Bibb Lettuce. International Journal of Vegetable Science. 2018;24: 122–136. doi: 10.1080/19315260.2017.1378787

[54] Yep B, Gale NV, Zheng Y. Aquaponic and Hydroponic Solutions Modulate NaCl-Induced Stress in Drug-Type *Cannabis sativa* L. Frontiers in Plant Science. 2020;11. Available: https://www.frontiersin.org/articles/10.3389/fpls.2020.01169 10.3389/fpls.2020.01169 PMC7424260 32849724

[55] Silberbush M, Ben-Asher J. The effect of NaCl concentration on NO3−, K+ and orthophosphate-P influx to peanut roots. Scientia Horticulturae. 1989;39: 279–287. doi: 10.1016/0304-4238(89)90121-0

[56] Freitas WE de S, Oliveira AB de, Mesquita RO, Carvalho HH de, Prisco JT, Gomes-Filho E. Sulfur-induced salinity tolerance in lettuce is due to a better P and K uptake, lower Na/K ratio and an efficient antioxidative defense system. Scientia Horticulturae. 2019;257: 108764. doi: 10.1016/j.scienta.2019.108764

[57] Xu C, Mou B. Evaluation of Lettuce Genotypes for Salinity Tolerance. HortScience. 2015;50: 1441–1446. doi: 10.21273/HORTSCI.50.10.1441

[58] Devic E, Leschen W, Murray F, Little D c. Growth performance, feed utilization and body composition of advanced nursing Nile tilapia (*Oreochromis niloticus*) fed diets containing Black Soldier Fly (*Hermetia illucens*) larvae meal. Aquaculture Nutrition. 2018;24: 416–423. doi: 10.1111/anu.12573

[59] Zhou J s., Liu S s., Ji H, Yu H b. Effect of replacing dietary fish meal with black soldier fly larvae meal on growth and fatty acid composition of Jian carp (*Cyprinus carpio* var. Jian). Aquaculture Nutrition. 2018;24: 424–433. doi: 10.1111/anu.12574

