# Supplementary material for: Insect-based fish feed in decoupled aquaponic systems: Effect on lettuce production and resource use
Source: PLoS One. 2024 Jan 19;19(1):e0295811. doi: 10.1371/journal.pone.0295811 (PMC10798475; doi:10.1371/journal.pone.0295811)
Supplement: S1 Appendix — A brief description of the setup and management of the aquaculture subsystems, which generated the fish waste water used in the hydroponics units of the decoupled aquaponics systems, is provided. (DOCX) [file pone.0295811.s001.docx]

**S1 Appendix. Aquaculture subsystem.** A brief description of the setup and management of the aquaculture subsystems, which generated the fish waste water used in the hydroponics units of the decoupled aquaponics systems, is provided.

The aquaculture units of the decoupled aquaponic systems were run in six experimental recirculating aquaculture systems (RAS) at the Leibniz Institute of Freshwater Ecology and Inland Fisheries (IGB, Berlin, Germany). Each RAS was divided into four sections by perforated PVC boards: a fish rearing section (64 L), two consecutive fixed-bed biofilter sections (32 L each) equipped with a 10 cm thick filter sponge (1 × PPI 20 + 1 × PPI 30, Schaumstoff-Meister, Straelen, Germany) each and a moving-bed bioreactor (MBBR) section (32 L) filled with Hel-X biocarriers (HXF12KLL, Christian Stöhr). The space in front of the filter sponge of the first fixed-bed biofilter functioned as sedimentation and collection area for the solids. Water recirculation from MBBR to the fish rearing section was ensured by an air lift and air stones in the fish rearing section as well as the MBBR provided oxygenation and enabled mixing of the biocarriers.

The RAS were stocked with juvenile Nile tilapia (*Oreochromis niloticus*) that were reared for 56 days on two experimental diets differing by the type of protein ingredient: an entirely FM-based diet and an entirely BSF meal-based diet. In brief, fish were hand fed twice per day at 2.5% of the initial mean biomass of all six RAS and rations were adjusted on days 14 and 38 of the fish trial, again according to the mean biomass of all six RAS. Consequently, all replicates received the same daily as well as total feed ratio over the course of the fish trial.

Physicochemical water parameters (dissolved oxygen, temperature, pH, electrical conductivity) were measured daily in the fish tanks (HQ40d, Hach Lange, Berlin, Germany; pH/Cond 740i, WTW, Weilheim in Oberbayern, Germany). A mean daily water exchange of 8.4% of the RAS volume was performed by siphoning out water and the accumulated solids from the sedimentation chamber. Fish waste water used in the hydroponics units of the aquaponics treatments (FM and BSF) was collected from the biofilter section of each RAS during each water exchange process for three consecutive days prior to the preparation of the nutrient solutions for the experiment (this was done three times during the experiment, prior to day 0, to day 14 and finally to day 27). On each of the three consecutive days, 15 L of fish waste water was collected per replicate of each treatment and accordingly, 135 L were collected within the three-day collection period per treatment (15 L * 3 (replicates per treatment) * 3 (consecutive days). The fish waste water from all replicates was mixed per treatment and three-day time period and stored individually in tanks (for FM and BSF separately) oxygenated with compressed air for further preparation of the nutrient solutions.

| ***Growth parameters*** *^1^* | **FM** | | | **BSF** | | |
| --- | --- | --- | --- | --- | --- | --- |
| Initial mean body weight (g) | 23.4 | ± | 0.50 | 24.1 | ± | 0.20 |
| Final mean body weight (g) | 70.2 | ± | 1.50 | 59.9 | ± | 1.60 |
| Final biomass (kg) | 1.40 | ± | 0.02 | 1.19 | ± | 0.03 |
| Final stocking density (kg m^-3^) | 21.60 | ± | 0.45 | 18.42 | ± | 0.49 |
| Total feed input (g) | 882 |  |  | 882 |  |  |
| Survival (%) | 100 |  |  | 100 |  |  |
| FCR | 0.9 | ± | 0.0 | 1.2 | ± | 0.1 |
| ***Water parameters*** ^2^ |  |  |  |  |  |  |
| Dissolved oxygen (mg L^-1^) | 6.9 | ± | 0.4 | 7.1 | ± | 0.3 |
| Temperature (°C) | 26.4 | ± | 0.2 | 26.4 | ± | 0.5 |
| pH | 7.4 | ± | 0.7 | 7.6 | ± | 0.2 |
| Electrical conductivity (mS cm^-1^) | 0.96 | ± | 0.04 | 0.95 | ± | 0.04 |

**Table S1 Appendix. Summary of juvenile Nile tilapia growth and water parameter in recirculating aquaculture subsystems of the decoupled aquaponics system**.

FM: fish meal-based diet. BSF: Black Soldier Fly meal-based diet. Values represent means ± standard deviations: ^1^ n = 3; ^2^ n = 168.
