# Supplementary material for: Insect-based fish feed in decoupled aquaponic systems: Effect on lettuce production and resource use
Source: PLoS One. 2024 Jan 19;19(1):e0295811. doi: 10.1371/journal.pone.0295811 (PMC10798475; doi:10.1371/journal.pone.0295811)
Supplement: S1 Table — (DOCX) [file pone.0295811.s002.docx]

| **Sampling*** | **Treatment** | **NO_3_-N** | **NH_4_-N** | **P** | **K** | **Ca** | **Mg** | **S** |
| --- | --- | --- | --- | --- | --- | --- | --- | --- |
| Day 0 | HP | 155.0 ± 0.8 | 7.5 ± 0.2 | 46.5 ± 0.3 | 223.0 ± 0.4 | 177.2 ± 3.0 | 41.5 ± 0.2 | 84.2 ± 0.2 |
|  | FM | 152.5 ± 1.6 | 4.4 ± 0.2 | 47.9 ±0.0 | 221.4 ± 1.5 | 163.3 ± 1.7 | 37.8 ± 0.0 | 68.8 ± 0.3 |
|  | BSF | 153.1 ± 1.0 | 4.1 ± 0.0 | 46.1 ± 0.2 | 220.5 ± 2.8 | 166.7 ± 2.6 | 39.5 ± 0.1 | 67.7 ± 0.2 |
| Day 6 | HP | 170.2 ± 2.4 | 7.1 ± 0.1 | 46.6 ± 0.3 | 235.9 ± 3.1 | 185.5 ± 1.0 | 43.6 ± 0.2 | 89.3 ± 0.4 |
|  | FM | 162.9 ± 3.4 | 3.4 ± 0.2 | 48.0 ± 0.5 | 231.2 ± 4.0 | 174.3 ± 2.3 | 39.7 ± 0.4 | 74.4 ± 0.1 |
|  | BSF | 165.6 ± 1.7 | 3.9 ± 0.1 | 47.2 ± 0.3 | 232.3 ± 3.8 | 178.1 ± 1.6 | 41.8 ± 0.4 | 73.9 ± 0.6 |
| Day 15 | HP | 165 ± 0.7 | 5.7 ± 0.1 | 49.3 ± 0.6 | 222.8 ± 0.8 | 184.5 ± 1.8 | 43.8 ± 0.7 | 80.2 ± 0.5 |
|  | FM | 157.6 ± 2.4 | 3.3 ± 0.1 | 53.1 ± 0.7 | 208.1 ± 0.5 | 182.2 ± 1.2 | 30.8 ± 0.4 | 67.6 ± 0.3 |
|  | BSF | 159.5 ± 1.1 | 2.9 ± 0.1 | 53.9 ± 0.8 | 212.9 ± 0.8 | 185.9 ± 1.4 | 33.8 ± 0.6 | 66.4 ± 0.5 |
| Day 21 | HP | 180.3 ± 3.3 | 0.2 ± 0.02 | 51.9 ± 0.2 | 228.5 ± 4.1 | 195.9 ± 1.7 | 48.1 ± 0.4 | 85.9 ± 0.3 |
|  | FM | 164.4 ± 0.9 | 0.3 ± 0.02 | 56.3 ± 0.3 | 216.4 ± 6.5 | 192.9 ± 1.1 | 33.8 ± 0.3 | 72.8 ± 0.5 |
|  | BSF | 162.6 ± 2.3 | 0.3 ± 0.02 | 56.0 ± 1.4 | 218.5 ± 4.2 | 194.2 ± 4.4 | 36.3 ± 0.1 | 70.2 ± 1.8 |
| Day 26 | HP | 164.4 ± 3.8 | 7.1 ± 0.03 | 44.8 ± 0.1 | 187.8 ± 1.0 | 181.0 ± 0.4 | 41.2 ± 0.2 | 81.9 ± 0.2 |
|  | FM | 155.0 ± 1.9 | 4.0 ± 0.1 | 48.3 ± 01 | 184.7 ± 0.8 | 181.6 ± 1.2 | 29.8 ± 0.04 | 69.8 ± 0.3 |
|  | BSF | 161.5 ± 1.4 | 4.1 ± 0.02 | 46.6 ± 0.1 | 185.0 ± 0.3 | 184.6 ± 0.2 | 31.9 ± 0.04 | 68.2 ± 0.2 |
| Day 33 | HP | 165.2 ± 1.1 | 0.5 ± 0.1 | 44.0 ± 0.3 | 167.6 ± 0.3 | 195.2 ± 0.9 | 43.4 ± 0.3 | 88.6 ± 0.5 |
|  | FM | 153.0 ± 3.3 | 0.7 ± 0.1 | 48.0 ± 0.4 | 166.1 ± 1.3 | 195.1 ± 0.9 | 31.3 ± 0.1 | 75.5 ± 0.3 |
|  | BSF | 159.9 ± 0.3 | 0.5 ± 0.02 | 46.2 ± 0.4 | 167.0 ± 2.4 | 198.5 ± 2.2 | 33.7 ± 0.4 | 73.8 ± 0.8 |

**S1 Table. Macronutrient concentrations (N, P, K, Ca, Mg, and S, mg L^-1^) in the nutrient solutions.** Lettuce production in the conventional hydroponic system (HP) and decoupled aquaponic systems reusing fish waste water from tilapia culture fed with fish meal-based diet (FM) and Black Soldier Fly meal-based diet (BSF).

* Water samples were collected for characterising the fresh nutrient solutions on days 0, 15 and 26, and every week after the water exchange procedure in all replicates on days 6, 21, and 33.
